# Design and fabrication of high-performance diamond triple-gate field-effect transistors

**DOI:** 10.1038/srep34757

**Published:** 2016-10-06

**Authors:** Jiangwei Liu, Hirotaka Ohsato, Xi Wang, Meiyong Liao, Yasuo Koide

**Affiliations:** 1 International Center for Young Scientists, National Institute for Materials Science (NIMS), 1-1 Namiki, Tsukuba, Ibaraki 305-0044, Japan; 2 Nanofabrication Platform, NIMS, 1-2-1 Sengen, Tsukuba, Ibaraki 305-0047, Japan; 3 Optical and Electronic Materials Unit, NIMS, 1-1 Namiki, Tsukuba, Ibaraki 305-0044, Japan; 4 Research Network and Facility Services Division, NIMS, 1-2-1 Sengen, Tsukuba, Ibaraki, 305-0047, Japan

## Abstract

The lack of large-area single-crystal diamond wafers has led us to downscale diamond electronic devices. Here, we design and fabricate a hydrogenated diamond (H-diamond) triple-gate metal-oxide-semiconductor field-effect transistor (MOSFET) to extend device downscaling and increase device output current. The device’s electrical properties are compared with those of planar-type MOSFETs, which are fabricated simultaneously on the same substrate. The triple-gate MOSFET’s output current (174.2 mA mm^−1^) is much higher than that of the planar-type device (45.2 mA mm^−1^), and the on/off ratio and subthreshold swing are more than 10^8^ and as low as 110 mV dec^−1^, respectively. The fabrication of these H-diamond triple-gate MOSFETs will drive diamond electronic device development forward towards practical applications.

Semiconductor diamond has some extraordinary physical properties, including a wide band gap energy (5.47 eV), a low dielectric constant (5.7), a theoretical high breakdown field (10 MV cm^−1^), high carrier saturation velocity (1.5–2.7 × 10^7^ and 0.85–1.2 × 10^7^ cm s^−1^ for electrons and holes, respectively)^1^,^2^, highest thermal conductivity (22 W cm^−1^ K^−1^), and high carrier mobility (4500 and 3800 cm^2^ V^−1^ s^−1^ for electrons and holes, respectively)^3^. According to the figures of merit quoted for diamond and other semiconductor materials^4^, diamond-based electronic devices have the highest power-frequency product, the highest thermal limitation, and the lowest power-loss at high frequencies. Diamond is therefore considered the most suitable material for fabrication of next-generation high-power, high-frequency, high-temperature, low-power-loss, and energy-saving electronic devices^5^. Because the activation energies of diamond dopants are much higher than the room temperature thermal energy, many diamond electronic devices have been fabricated on hydrogenated diamond (H-diamond) channel layers^6^,^7^,^8^,^9^,^10^,^11^. The H-diamond can accumulate holes on its surface with a sheet hole density (*p*_*sheet*_) of 10^12^–10^13^ cm^−2^. In fact, exposure of H-diamond to a NO_2_ ambient^12^ or annealing of oxygen-terminated diamond in an NH_3_ ambient^13^ can produce *p*_*sheet*_ for H-diamond of as high as 10^14^ cm^−2^. Recently, fabrication processes for H-diamond metal-oxide-semiconductor field-effect transistors (MOSFETs) have been developed. The maximum drain-source current (*I*_*DS,max*_) of a MOSFET at room temperature fabricated on NO_2_-treated H-diamond^14^ was as much as −1.35 A mm^−1^ under conditions of gate-source voltage (*V*_*GS*_), drain-source voltage (*V*_*DS*_), and gate length (*L*_*G*_) of −5 V, −12 V, and 0.4 μm, respectively. The cut-off frequency of the device was more than 10 GHz over a wide *V*_*GS*_ range of approximately 10.0 V. In addition, the operational performance of H-diamond-based MOSFETs was comparable to that of SiC- or GaN-based MOSFETs in high temperature (400 °C) and high voltage (500 V) operation^15^.

While these H-diamond-based MOSFETs showed excellent electrical properties, the absence of large-area single-crystal diamond wafers has hindered their development for widespread practical applications. This issue has led us to downscale diamond electronic devices. In our previous studies^16^,^17^, downscaled H-diamond MOSFETs were fabricated by eliminating the interspacing between the source/drain and gate contacts (*L*_*S/D-G*_). The on-resistance (*R*_*ON*_) of the H-diamond MOSFET without *L*_*S/D-G*_ (29.7 Ω mm) was considerably lower than the corresponding device with *L*_*S/D-G*_ (208.4 Ω mm). The device’s current output and extrinsic transconductance (*g*_*m*_) of the former were also around seven times higher than those of the latter. Other studies that focused on downscaling of the *L*_*G*_ for H-diamond MOSFETs were also reported^18^. The shortest *L*_*G*_ for the single crystalline H-diamond MOSFETs were downscaled to be around 100 nm. Recently, triple-gate MOSFET architecture has been developed in the Si-, InGaAs-, and GaN-based MOSFETs to extend device downscaling, reduce leakage current, and control device short channel effects^19^,^20^,^21^,^22^,^23^,^24^,^25^,^26^,^27^. Also, because the triple-gate MOSFET can allow carriers to travel in both its planar and lateral sides, the device current output is much higher than that of a planar-type device with the same area. The fabrication of H-diamond triple-gate MOSFETs is therefore promising for extension of device downscaling and enhancement of the device electrical properties.

Here, we describe the design and fabrication of H-diamond triple-gate MOSFETs on a single crystalline diamond substrate. The electrical properties of these devices are compared with those of planar-type MOSFETs. The absolute *I*_*DS,max*_ of the triple-gate MOSFET is 174.2 mA mm^−1^, which is much higher than the 45.2 mA mm^−1^ value of the planar-type device. In addition, the on/off ratio and the subthreshold swing (SS) of the H-diamond triple-gate MOSFET are higher than 10^8^ and as low as 110 mV dec^−1^, respectively.

## Results

### Fin-patterned H-diamond and triple-gate MOSFET fabrication

**Figure 1:**
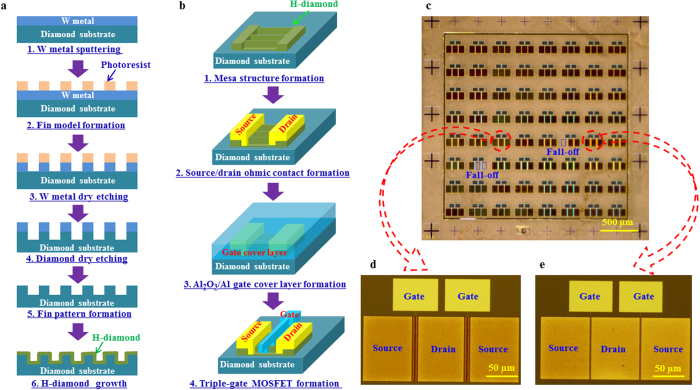
Fabrication of fin-patterned H-diamond and triple-gate MOSFETs. (**a,b**) Fabrication routines for the fin-patterned H-diamond and triple-gate MOSFETs, respectively. (**c**) Top view of the entire sample surface. Three ohmic contacts fell off during the fabrication process. (**d**) Top view of two triple-gate H-diamond MOSFETs. (**e**) Top view of two planar-type H-diamond MOSFETs.

**Figure 2:**
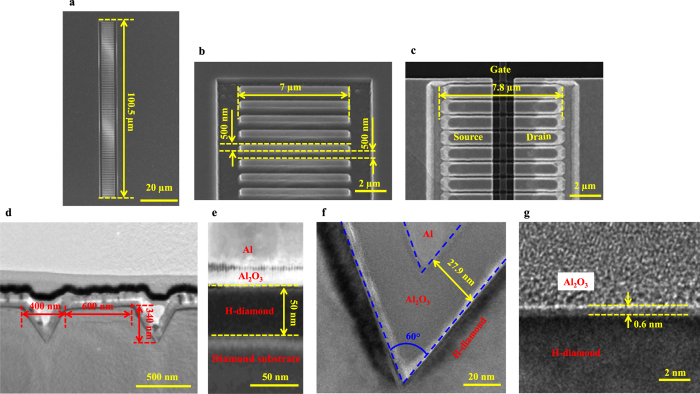
Surface and interface morphologies. (**a,b**) SEM images of the fin-patterned diamond substrate. (**c**) SEM image of the triple-gate MOSFET. (**d–g**) Interfacial TEM images of the triple-gate H-diamond MOSFET.

**Figure 3:**
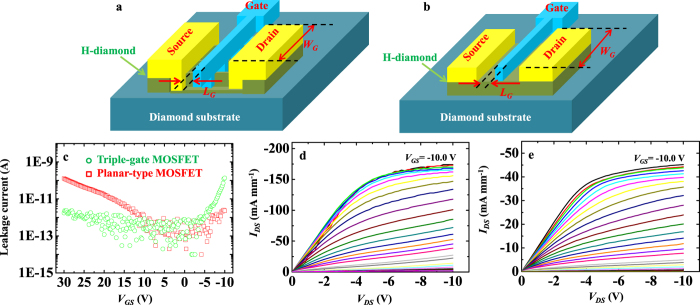
Electrical properties of the triple-gate and planar-type MOSFETs. (**a,b**) Schematic diagrams of the triple-gate and planar-type MOSFETs, respectively. (**c**) The *I*_*G,leak*_ curves for the triple-gate and planar-type MOSFETs. Green cycle and red square curves represent the *I*_*G,leak*_ of the triple-gate and planar-type MOSFETs, respectively. (**d,e**) *I*_*DS*_*-V*_*DS*_ characteristics for the triple-gate and planar-type MOSFETs, respectively. The *V*_*GS*_ is varied from −10.0 to 20.0 V in steps of +1.0 V.

**Figure 4:**
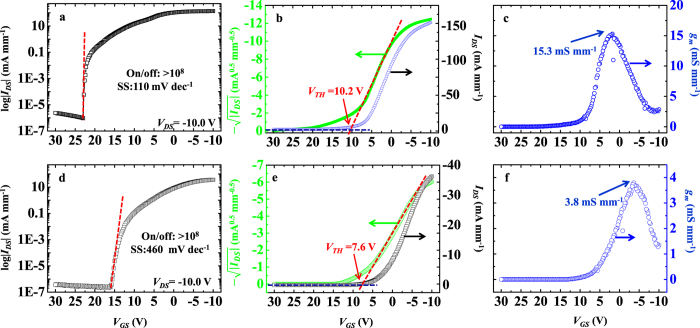
Transfer characteristics of *I*_*DS*_*–V*_*DS*_ for triple-gate and planar-type MOSFETs. (**a–c**) The log |*I*_*DS*_|*–V*_*GS*_, 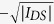
–*V*_*GS*_, and *g*_*m*_*–V*_*GS*_ characteristics of the triple-gate MOSFET, respectively. (**d–f**) The log |*I*_*DS*_|*–V*_*GS*_, 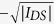
–*V*_*GS*_, and *g*_*m*_*–V*_*GS*_ characteristics of the planar-type MOSFET, respectively.

Figure 1 shows the fabrication process flows for (a) fin-patterned H-diamond MOSFETs and (b) triple-gate MOSFETs, (c) the top view of the entire sample surface, (d) the top view of two triple-gate H-diamond MOSFETs, and (e) the top view of two planar-type H-diamond MOSFETs. To form the fin patterns on the diamond (001) substrate, a tungsten (W) metal layer was first sputtered using an automatic sputtering system to cover the entire substrate surface [Fig. 1(a)-1]. The positive photoresist FEP-171 was then coated on the sample and exposed using an electron beam (EB) lithography system to form fin models [Fig. 1(a)-2]. After the photoresist was developed, the W metal and the diamond substrate at the photoresist-free area were dry-etched in SF_6_ and O_2_ ambients, respectively, using an inductively-coupled plasma reactive ion etching (ICP-RIE) system [Fig. 1(a)-3 and 4]. The residual W metal was cleaned again in the SF_6_ ambient to form fin patterns on the diamond surface [Fig. 1(a)-5]. Then, the H-diamond epitaxial layer was grown on the substrate by microwave plasma chemical vapour deposition (MPCVD) to form fin-patterned H-diamond [Fig. 1(a)-6].

After the formation of fin-patterned H-diamond, the triple-gate MOSFETs were then fabricated [Fig. 1(b)]. The fin-patterned H-diamond was first etched in an O_2_ ambient using a capacitively-coupled plasma RIE (CCP-RIE) system to form a mesa structure [Fig. 1(b)-1]. Palladium/titanium/gold (Pd/Ti/Au) ohmic contacts were then evaporated on the fin-patterned H-diamond to form the source/drain electrodes using an electron-gun (E-gun) evaporation system [Fig. 1(b)-2]. The aluminium oxide (Al_2_O_3_) gate insulator layer which has been used for the fabrication of high-performance diamond MOS devices in the previous reports^14^,^15^,^28^ and the aluminium (Al) gate electrode were then deposited to cover the entire sample surface by atomic layer deposition (ALD) and ultra-high-vacuum (UHV) sputtering techniques, respectively [Fig. 1(b)-3]. Then, the sample was coated with PMGI-SF6S/FEP-171 bilayer photoresists and exposed using the EB lithography system to form gate models. After development of the photoresists, the Al and Al_2_O_3_ layers on the photoresist-free areas were wet-etched using mixed Al etching acid and tetramethylammonium hydroxide (TMAH) solutions, respectively. Finally, the photoresists were lifted off in an N-methylpyrrolidone (NMP) solution, and the fabrication of the triple-gate H-diamond MOSFETs was complete [Fig. 1(b)-4]. Planar-type MOSFETs were also fabricated simultaneously with the triple-gate devices on the same diamond substrate. Figure 1(c) shows a top view of the entire surface of the sample. The total number of designed MOSFETs was 128. However, three ohmic contacts fell off during the fabrication process. The top views of triple-gate and planar-type H-diamond MOSFETs are shown in Fig. 1(d,f), respectively. The *L*_*G*_, *L*_*S/D-G*_, and gate width (*W*_*G*_) for these devices were 500 nm, 500 nm, and 100.5 μm, respectively.

### Surface and interface morphologies

Figure 2 shows scanning electron microscopy (SEM) [Fig. 2(a–c)] images of the fin-patterned diamond substrate and triple-gate MOSFET, and transmission electron microscopy (TEM) [Fig. 2(d–g)] images of interface of the triple-gate H-diamond MOSFET. As shown in the SEM images [Fig. 2(a,b)], the total width of the diamond fin pattern and the fin length are 100.5 and 7 μm, respectively. Both the fin width and the interspacing between fins are 500 nm. The fin height was confirmed using a 3D-measurement laser microscope to be 500 nm. Obvious gate, source, and drain contacts for the H-diamond triple-gate MOSFET can be seen in Fig. 2(c). After H-diamond epitaxial layer growth by the MPCVD technique, the fin length and width increased to 7.8 μm and 600 nm, respectively. The interspacing between fins and the fin height both decreased to 400 and 340 nm, respectively [Fig. 2(d)]. The H-diamond epitaxial layer thickness is approximately 50 nm [Fig. 2(e)]. Figure 2(f) shows a high-resolution TEM image for the zoom of the left adjacent fins in the Fig. 2(d). The angle between two adjacent fins is 60°. The two inclined active planes of each fin in the triple-gate MOSFETs are the 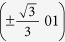 sides. The equivalent *W*_*G*_ for the triple-gate MOSFET can be calculated to be 139.6 μm. The ALD-Al_2_O_3_ layer thickness is approximately 27.9 nm, which is in good agreement with the measurement results obtained using an ellipsometer system. An interfacial layer with thickness of around 0.6 nm exists between H-diamond and Al_2_O_3_ [Fig. 2(g)], and a similar layer is also observed at the AlN/H-diamond interface^29^. The origins of these layers are still under discussion at present, however, the layers are possibly a result of reactions between the oxides or nitrides and the surface adsorbates on the H-diamond epitaxial layer^30^.

### Electrical properties of triple-gate and planar-type MOSFETs

Figure 3 shows (a) and (b) schematic diagrams of the triple-gate and planar-type H-diamond MOSFETs, respectively, (c) gate leakage current (*I*_*G,leak*_) for the triple-gate and planar-type MOSFETs, and (d) and (e) drain-source current versus voltage (*I*_*DS*_-*V*_*DS*_) characteristics for the triple-gate and planar-type MOSFETs, respectively. Both triple-gate and planar-type MOSFETs have the same *L*_*G*_, *W*_*G*_, and *L*_*S/D-G*_. The difference for them is the existence of fin-patterns on the diamond substrate for the triple-gate MOSFET. The *I*_*G,leak*_ curves for the MOSFETs were measured with the *V*_*GS*_ changing from 30.0 to −10.0 V. At the *V*_*GS*_ of −10.0 V, the holes are accumulated at the Al_2_O_3_/H-diamond interface and the MOSFETs are at on-states. The *I*_*G,leak*_ of the triple-gate MOSFET is 1.4 × 10^−10^ A, which is higher than that of the planar-type one of 2.3 × 10^−12^ A. This is possibly ascribed to the longer equivalent *W*_*G*_ and rougher etching surface for the triple-gate MOSFET than those for the planar-type one. The *I*_*G,leak*_ density for the planar-type MOSFET can be calculated to be 4.6 × 10^−6^ A cm^−2^ using the *I*_*G,leak*_ divided by area of gate electrode (5.025 × 10^−7^ cm^2^). It is one order higher than that of the Al_2_O_3_/H-diamond MOS capacitor^31^ of 1.1 × 10^−7^ A cm^−2^. Since the fabrication process for MOSFET is more complicated than that for MOS capacitor, the device damage during fabrication for the former is more serious than that for the latter, which possibly leads to the higher *I*_*G,leak*_ for the MOSFET. At the *V*_*GS*_ of 30.0 V, holes are difficult to be accumulated at the Al_2_O_3_/H-diamond interface and MOSFETs are at off-states. The *I*_*G,leak*_ for the triple-gate MOSFET is 1.8 × 10^−12^ A, which is lower than that for the planar-type one of 1.3 × 10^−10^ A.

The *V*_*GS*_ is varied from −10.0 to 20.0 V in steps of +1.0 V for measurement of the *I*_*DS*_–*V*_*DS*_ characteristics of the triple-gate and planar-type MOSFETs [shown in Fig. 3(d,e), respectively]. The *I*_*DS*_ for the planar-type MOSFET was normalized with the *W*_*G*_ of 100.5 μm. That for the triple-gate MOSFET was normalized with its equivalent *W*_*G*_ of 139.6 μm. Both MOSFETs show obvious *p*-type channel and pinch-off characteristics. There are also good linear relationships between *I*_*DS*_ and low *V*_*DS*_ for both devices, which indicate good ohmic contact between the Pd/Ti/Au and H-diamond channel layers. The absolute *I*_*DS,max*_ for the triple-gate MOSFET is 174.2 mA mm^−1^, which is much higher than the value of 45.2 mA mm^−1^ obtained for the planar-type device. The value of *R*_*ON*_ can be extracted from the linear region of the *I*_*DS*_–*V*_*DS*_ characteristics, and is 31.9 and 98.0 Ω mm for the triple-gate and planar-type MOSFETs, respectively. The *R*_*ON*_ for the triple-gate H-diamond MOSFET is composed of the fin pattern channel resistance beneath the Al_2_O_3_ insulator (*R*_*CH*_), the fin pattern H-diamond surface resistance with the *L*_*S/D-G*_ of 500 nm (2*R*_*SD*_), and the Pd/Ti/Au ohmic contact resistance (2*R*_*C*_). Because 2*R*_*C*_ is much smaller than *R*_*CH*_ and 2*R*_*SD*_, it can be neglected here^32^. By combining the *R*_*ON*_ value of another two triple-gate MOSFETs with the *L*_*S/D-G*_ of 1.0 and 2.0 μm (The electrical properties of them are shown in Fig. S1 of the Supplementary Information), *R*_*CH*_ and 2*R*_*SD*_ for the triple-gate MOSFET can be deduced to be 23.8 and 8.1 Ω mm, respectively.

The transfer characteristics that correspond to the *I*_*DS*_–*V*_*DS*_ curves are shown in Fig. 4. The on/off ratio of the triple-gate MOSFET is higher than 10^8^ [Fig. 4(a)], and is the same level as that of the planar-type device [Fig. 4(d)]. The SS is an important parameter for evaluation of MOSFET power consumption, and is defined as the inverse slope of log |*I*_*DS*_| versus *V*_*GS*_. The SS is 110 mV dec^−1^ for the triple-gate MOSFET at a *V*_*DS*_ of −10.0 V [Fig. 4(a)]. This value is much lower than that of the planar-type device of 460 mV dec^−1^ [Fig. 4(d)]. There is also a relationship between the SS and the interfacial trap charge density (*D*_*it*_) of 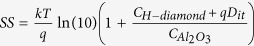. Here, *k*, *T*, *q*, *C*_*H-diamond*_ and 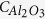 are Boltzmann’s constant (8.62 × 10^−5^ eV K^−1^), room temperature (298.15 K), the elementary charge (1.6 × 10^−19^ C), the capacitance of the H-diamond, and the capacitance of the Al_2_O_3_ layer, respectively. 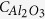 can be calculated using the equation 
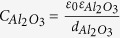
 to be 0.171 μF cm^−2^, where *ε*_0_, 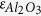, and 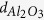 are the dielectric constant of a vacuum (8.85 × 10^−12^ F m^−1^), the dielectric constant of Al_2_O_3_ (5.4)^31^, and the thickness of the Al_2_O_3_ layer (27.9 nm), respectively. If *C*_*H-diamond*_ can be neglected in the deep-subthreshold region, the *D*_*it*_ values for the triple-gate and planar-type MOSFETs are then calculated to be 8.95 × 10^11^ and 7.14 × 10^12^ eV^−1^ cm^−2^, respectively. The threshold voltage (*V*_*TH*_) values of the MOSFETs can be determined based on 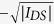 as functions of *V*_*GS*_, and are 10.2 ± 0.1 and 7.6 ± 0.1 V for the triple-gate and planar-type MOSFETs, respectively [Fig. 4(b,e)].

The following relationship exists between *R*_*ON*_, *V*_*TH*_, and effective mobility (*μ*_*eff*_): 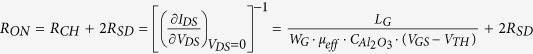. The values of *R*_*ON*_, *L*_*G*_, *W*_*G*_, 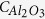, *V*_*GS*_, *V*_*TH*_, and 2*R*_*SD*_ for the triple-gate MOSFET are 31.9 Ω mm, 500 nm, 139.6 μm, 0.171 μF cm^−2^, −10.0 V, 10.2 ± 0.1 V, and 8.1 Ω mm, respectively. The *μ*_*eff*_ of the fin-patterned H-diamond channel layer can be calculated to be 6.1 ± 0.5 cm^2^ V^−1^ s^−1^. This is lower than the value for the planar-type device of 38.7 ± 0.5 cm^2^ V^−1^ s^−1^ that was reported previously^33^, and can possibly be attributed to the increased surface roughness at the etching area for the fin-patterned H-diamond channel layer. The extrinsic transconductance (*g*_*m*_) is determined based on the slope of the *I*_*DS*_–*V*_*GS*_ curve. The maximum *g*_*m*_ (*g*_*m,max*_) values for the triple-gate and planar-type MOSFETs are 15.3 ± 0.1 and 3.8 ± 0.1 mS mm^−1^, respectively [Fig. 4(c,f)].

## Discussion

The H-diamond triple-gate MOSFET has been fabricated and characterized to compare with those of the corresponding planar-type device, and these properties are summarized in Table 1. While the equivalent *W*_*G*_ of the triple-gate MOSFET is only 1.4 times longer than *W*_*G*_ for the planar-type device, the absolute *I*_*DS,max*_ for the former (174.2 mA mm^−1^) is around four times larger than the corresponding value for the latter (45.2 mA mm^−1^). This was confirmed again using another triple-gate MOSFET, as shown in Fig. S2 of the Supplementary Information. It was previously reported that the inclined H-diamond (111) plane had a higher *p*_sheet_ than the planar H-diamond (001) plane^34^. In this study, because each fin of the triple-gate MOSFET has two inclined (±
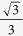
01) planes, it is natural to believe that the *p*_sheet_ of the fin-patterned H-diamond channel layer must be higher than that of the planar H-diamond (001) layer. This is possibly the reason for the higher *I*_*DS,max*_ and lower *R*_*ON*_ obtained for the triple-gate MOSFET in comparison to the theoretical values. The *I*_*DS,max*_ for the triple-gate MOSFET is still much lower than that of the NO_2_-treated H-diamond-based MOSFET (−1.35 A mm^−1^)^14^, which possibly attributed to the higher hole density for the NO_2_-treated H-diamond channel layer and the poor crystalline quality of the large-area diamond wafer^35^ used in this study. We have also attempted to fabricate the triple-gate MOSFET without the *L*_*S/D-G*_. The electrical properties of the resulting device are shown in Fig. S3 of the Supplementary Information. While the *I*_*DS,max*_ of this device is as much as 251.4 mA mm^−1^, the output current cannot be controlled well with changes in *V*_*GS*_. In the triple-gate MOSFET without the *L*_*S/D-G*_, the Al_2_O_3_/Al gate layers also cover the source/drain ohmic contacts. During the Al_2_O_3_/Al etching process [Fig. 1(b)-4], strong damage may possibly occur at the edge area, which could lead to high gate leakage and poor electrical properties for the resulting MOSFET. The on/off ratios of both the triple-gate and planar-type MOSFETs are higher than 10^8^, and are thus high enough for practical applications. *D*_*it*_ for the triple-gate MOSFET (8.95 × 10^11^ eV^−1^ cm^−2^) is lower than that for the planar-type device (7.14 × 10^12^ eV^−1^ cm^−2^), which leads to the SS of the triple-gate MOSFET (110 mV dec^−1^) being much lower than that of the planar-type MOSFET (460 mV dec^−1^). The *V*_*TH*_ for the triple-gate MOSFET is larger than that for the planar-type MOSFET, which is possibly attributed to the higher *p*_sheet_ for the fin-pattern H-diamond channel layer. Both *V*_*TH*_ values are much higher than zero for the MOSFETs, which indicates that the devices operate with depletion modes. Recently, control conditions for the fabrication of depletion/enhancement mode H-diamond MOSFETs have been verified^36^. Therefore, it is promising for fabrication of enhancement-mode H-diamond triple-gate MOSFETs in future work. The *g*_*m,max*_ (15.3 ± 0.1 mS mm^−1^) of the triple-gate MOSFET is much higher than that of the planar-type device (3.8 ± 0.1 mS mm^−1^).

In conclusion, the H-diamond triple-gate MOSFETs have been fabricated on a single crystalline diamond substrate. The electrical properties of these devices are compared with those of planar-type MOSFETs. The absolute *I*_*DS,max*_ of the triple-gate MOSFET is 174.2 mA mm^−1^, which is much higher than the 45.2 mA mm^−1^ value of the planar-type device. In addition, the on/off ratio and the SS of the H-diamond triple-gate MOSFET are higher than 10^8^ and as low as 110 mV dec^−1^, respectively. The fabrication of these high-performance H-diamond triple-gate MOSFETs will drive the development of diamond electronic devices forward towards practical applications.

## Methods

### Fin-patterned H-diamond fabrication

The CVD single crystalline diamond (001) substrate, with dimensions of 5.0 × 5.0 × 0.3 mm was purchased from EDP Corp. It was cleaned in a mixed acid solution (H_2_SO_4_ and HNO_3_ with a volume ratio of 1:1) for 3 h at 300 °C. The W metal was sputtered on the diamond substrate at 300 W in an Ar gas ambient using an automatic sputtering system (JSP-8000, ULVAC, Kanagawa, Japan). The W layer thickness and sputtering time were 200 nm and 30 min, respectively. The W/diamond sample was coated with FEP-171 positive photoresist using a spin-coater with a rotation rate and time of 5000 rpm and 1 s, respectively. The baking temperature and time for the FEP-171 photoresist were 120 °C and 2 min, respectively. After exposure using the EB lithography system (ELS-7000, Elionix, Tokyo, Japan), the sample was developed in a TMAH solution for 1.5 min. The W metal was then etched via a Bosch process with SF_6_ and C_4_F_8_ gases using the ICP-RIE dry etching system (MUC-21, Sumitomo Precision Products, Hyogo, Japan). The SF_6_ and C_4_F_8_ flow rates were 75 and 60 sccm, respectively, and their plasma powers were 175 and 150 W, respectively. The diamond at the photoresist-free area was etched using the same equipment in an O_2_ gas ambient. The etching power, the O_2_ flow rate, the chamber pressure, and the etching time were 400 W, 10 sccm, 0.5 Pa, and 25 min, respectively. After cleaning of the residual W, the fin-patterned diamond substrate was formed. Then, the H-diamond epitaxial layer was grown using the MPCVD system (AX5200S, Seki Technotron Corp., Tokyo, Japan). Before growth commenced, the fin-patterned diamond substrate was annealed in the MPCVD chamber at 1000 °C for 20 min to clean off any surface contamination. The growth temperature, time, and chamber pressure for the H-diamond epitaxial layer were 900–940 °C, 20 min, and 80 Torr, respectively. The H_2_ and CH_4_ flow rates were 500 and 0.5 sccm, respectively.

### Triple-gate MOSFET fabrication

The fabrication of the triple-gate Al_2_O_3_/H-diamond MOSFETs was based on a combination of EB lithography, CCP-RIE dry etching, E-gun evaporation, ALD, UHV sputtering, wet etching, and lift-off techniques. The PMGI-SF6S/FEP-171 bilayer photoresists were sequentially coated on the fin-patterned H-diamond substrate. The baking conditions for the FEP-171 layer were given above. The baking temperature and time for the PMGI-SF6S layer were 180 °C and 5 min, respectively. The H-diamond channel layer was etched in an O_2_ ambient at a pressure of 10 Pa using the CCP-RIE system (RIE-200NL, Samco, Kyoto, Japan) to form the mesa structure. The plasma power and the etching time were 50 W and 1.5 min, respectively. The Pd/Ti/Au ohmic contact was formed using the E-gun evaporation system (RDEB-1206K, R-DEC Co. Ltd., Ibaraki, Japan), where the Pd metal layer was evaporated first to contact with the fin-patterned H-diamond surface. The Pd, Ti, and Au layer thicknesses were 10, 20, and 100 nm, respectively. The evaporation rates for these layers were 0.05, 0.05, and 0.2 nm s^−1^, respectively. The chamber pressure was in the 1.0–2.5 × 10^−5^ Pa range. The Al_2_O_3_ gate insulator and the Al gate electrode were deposited sequentially on the fin-patterned H-diamond channel layer using the ALD (SUNALE R-100B, Picosun, Tokyo, Japan) and UHV sputtering (LS-420R, Biemtron, Ibaraki, Japan) systems, respectively. The precursors for the ALD-Al_2_O_3_ layer were Al(CH_3_)_4_ and water vapour. The pulse and purge times for both precursors were 0.1 and 4.0 s, respectively. The deposition temperature was 120 °C. The plasma power, the chamber pressure, the Ar gas flow rate, and the deposition time for Al metal sputtering were 50 W, 0.3 Pa, 10 sccm, and 7 min, respectively. The Al metal was then wet etched using a mixed acid solution (volume ratio of H_3_PO_4_:HNO_3_:CH_3_COOH:H_2_O of 16:2:2:1) for 1 min. The Al_2_O_3_ insulator was wet etched using the TMAH solution for 10 min. The photoresists were removed using an NMP solution at room temperature for 3 h.

### Measurement system

The surface morphology of the fin-patterned diamond substrate was investigated using the SEM system (S-4800, Hitachi, Tokyo, Japan). The sample was prepared for TEM measurements using a focused ion beam-SEM (Xvision-200DB, SII Co., Chiba, Japan) system. TEM measurements were performed using the JEM-2100F system with an accelerating voltage of 200 kV. The fin pattern height was measured using a 3D-measurement laser microscopy system (OLS-4000, Olympus, Tokyo, Japan). The Al_2_O_3_ film thickness was measured using an ellipsometer system (MARY-102FM, Five Lab, Saitama, Japan). The electrical properties of the MOSFETs were measured using an MX-200/B prober (Vector Semiconductor Corp., Tokyo, Japan) and a B1500A parameter analyser (Agilent, Tokyo, Japan). The *I*_*G,leak*_ curves for the MOSFETs were obtained by measuring current-voltage relationships between the gate and source contacts. The *I*_*DS*_–*V*_*DS*_characteristics for the MOSFETs were obtained by measuring current-voltage relationships between the drain and source contacts with the change of *V*_*GS*_.

## Additional Information

**How to cite this article**: Liu, J. *et al*. Design and fabrication of high-performance diamond triple-gate field-effect transistors. *Sci. Rep.* **6**, 34757; doi: 10.1038/srep34757 (2016).

## Supplementary Material

Supplementary Information

## Figures and Tables

**Table 1:** Electrical properties of the triple-gate and planar-type MOSFETs.

	*|I*_*DS,max*_| (mA mm^−1^)	*R*_*ON*_(Ω mm)	On/off	*SS* (mV dec^−1^)	*D*_*it*_(eV^−1^ cm^−2^)	*V*_*TH*_(V)	*g*_*m,max*_(mS mm^−1^)
Triple-gate	174.2	31.9	>10^8^	110	8.95 × 10^11^	10.2 ± 0.1	15.3 ± 0.1
Planar-type	45.2	98.0	>10^8^	460	7.14 × 10^12^	7.6 ± 0.1	3.8 ± 0.1
