# Supplementary material for: Design and fabrication of high-performance diamond triple-gate field-effect transistors
Source: Sci Rep. 2016 Oct 6;6:34757. doi: 10.1038/srep34757 (PMC5052526; doi:10.1038/srep34757)
Supplement: Supplementary Information [file srep34757-s1.pdf]

Supplementary Information

Design and fabrication of high-performance diamond triple-gate field-effect transistors

Jiangwei Liu[1], Hirotaka Ohsato[2], Xi Wang[1], Meiyong Liao[3], and Yasuo Koide[4]

[1]*International Center for Young Scientists, National Institute for Materials Science (NIMS), 1-1 Namiki, Tsukuba, Ibaraki 305-0044, Japan*

[2]*Nanofabrication Platform, NIMS, 1-2-1 Sengen, Tsukuba, Ibaraki 305-0047, Japan*

[3]*Optical and Electronic Materials Unit, NIMS, 1-1 Namiki, Tsukuba, Ibaraki 305-0044, Japan*

[4]*Research Network and Facility Services Division, NIMS, 1-2-1 Sengen, Tsukuba, Ibaraki, 305-0047, Japan*

Correspondence and requests for materials should be addressed to J. L. (email: liu.jiangwei@nims.go.jp)

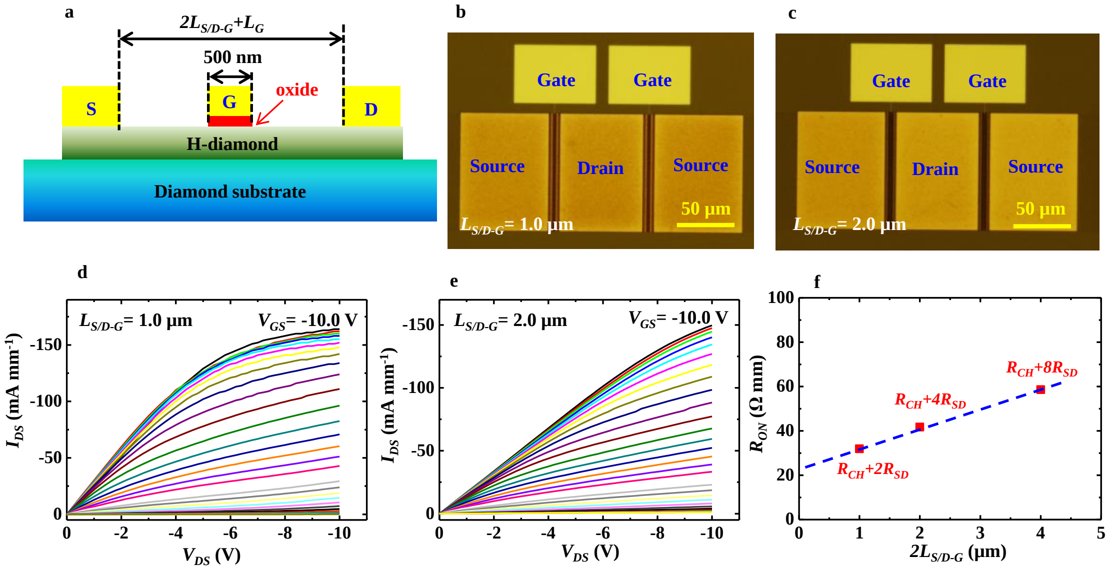

Figure S1| Electrical properties of triple-gate H-diamond MOSFETs with interspacing between source/drain and gate ($L_{S/D-G}$) of 1.0 and 2.0 µm. (a) Schematic of the cross-sectional structure for the MOSFETs. The gate length (L_G) is 500 nm. The interspacing between source and drain is $2L_{S/D-G} + L_G$. (b) and (c) Top views of two triple-gate MOSFETs with $L_{S/D-G}$ of 1.0 and 2.0 µm, respectively. (d) and (e) Drain-source current versus voltage (I_{DS}–V_{DS}) characteristics for the triple-gate MOSFETs with $L_{S/D-G}$ of 1.0 and 2.0 µm, respectively. The gate-source voltage (V_{GS}) is varied from −10.0 to 20.0 V in steps of +1.0 V. (f) On-resistance (R_{ON}) as a function of $2L_{S/D-G}$.

The absolute maximum I_{DS} ($I_{DS,max}$) normalized with equivalent gate width (W_G) of 139.6 µm are 164.0 and 149.7 mA mm^{-1} for the triple-gate MOSFETs with $L_{S/D-G}$ of 1.0 and 2.0 µm, respectively. These values are slightly lower than that (174.2 mA mm^{-1}) of the MOSFET in the manuscript with $L_{S/D-G}$ of 500 nm. The R_{ON} values for the triple-gate MOSFETs with $L_{S/D-G}$ of 1.0 and 2.0 µm are 41.7 and 58.6 Ω mm, respectively. If the surface resistance of the triple-gate MOSFET with $L_{S/D-G}$ of 500 nm is $2R_{SD}$, then the corresponding values for the MOSFETs with $L_{S/D-G}$ of 1.0 and 2.0 µm are considered to be $4R_{SD}$ and $8R_{SD}$, respectively. Based on the R_{ON} as a function $2L_{S/D-G}$, the R_{CH} can be deduced to be 23.8 Ω mm. Therefore, the $2R_{SD}$ for the triple-gate MOSFET with $L_{S/D-G}$ of 500 nm can be calculated to be 8.1 Ω mm.

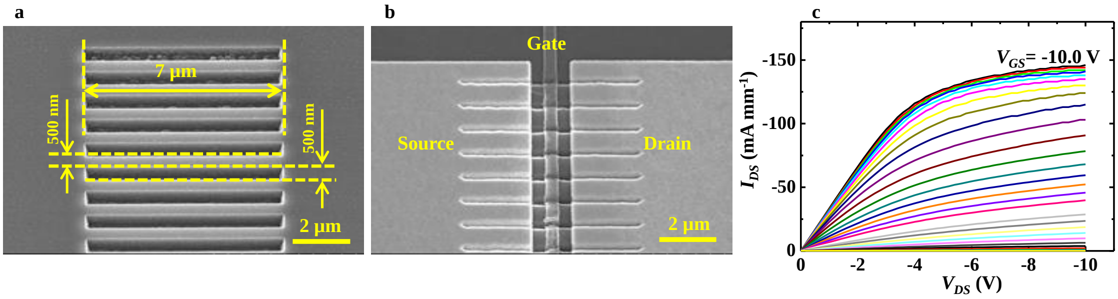

Figure S2| Triple-gate MOSFET fabricated on a second fin-patterned diamond substrate. (a) SEM image of the second fin-patterned diamond substrate. The fin length is 7 µm. Both the fin width and the interspacing between fins are 500 nm. (b) SEM image of the triple-gate MOSFET formed on the second fin-patterned diamond substrate. Both L_G and $I_{S/D\text{-}G}$ are 500 nm. (d) I_{DS}–V_{DS} characteristics of the triple-gate MOSFET. V_{GS} is varied from −10.0 to 20.0 V in steps of +1.0 V.

We have fabricated two fin patterns on the diamond substrate via etching processes. The SEM images of the first pattern are shown in the main manuscript in Fig. 2(a) and (b). The corresponding image of the second pattern is shown in Fig. S2(a). On the fin-patterned diamond shown in Fig. 2(b), the edge of the diamond substrate is etched. On the fin-patterned diamond shown in Fig. S2(a), the corresponding edge is not etched. The absolute $I_{DS,max}$ of the triple-gate MOSFET normalized with the equivalent W_G shown in Fig. S2(c) is 145.6 mA mm^{-1}, which is lower than that of the device in the main manuscript of 174.2 mA mm^{-1}. However, both values are much larger than that (45.2 mA mm^{-1}) of the planar-type device.

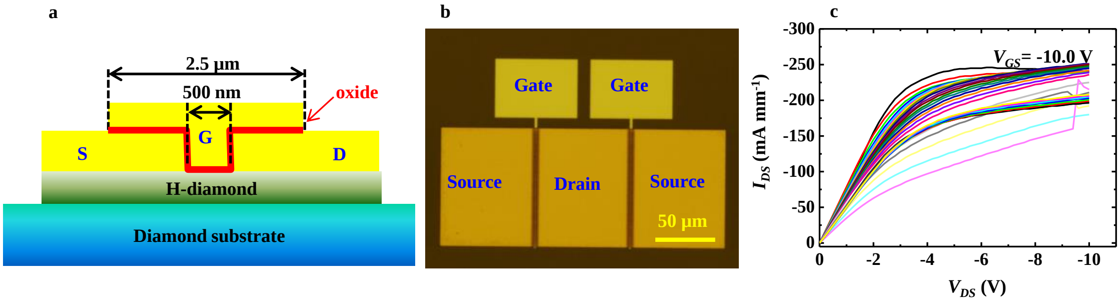

Figure S3| Triple-gate H-diamond MOSFET without $L_{S/D\text{-}G}$. (a) Schematic of the cross-sectional structure. The device has a T-shaped gate electrode with top and bottom lengths of 2.5 μm and 500 nm, respectively. (b) Top view of two triple-gate MOSFETs without $L_{S/D\text{-}G}$. (c) I_{DS}–V_{DS} characteristics of the triple-gate MOSFET without $L_{S/D\text{-}G}$. V_{GS} is varied from −10.0 to 20.0 V in steps of +1.0 V.

The absolute $I_{DS,max}$ of the triple-gate MOSFET without $L_{S/D\text{-}G}$ is 251.4 mA mm^{-1}, which is larger than that (174.2 mA mm^{-1}) of the triple-gate MOSFET with $L_{S/D\text{-}G}$ of 500 nm in the original manuscript. The R_{ON} of the device is only 13.6 Ω mm, which is lower than that of the triple-gate MOSFET with $L_{S/D\text{-}G}$ of 500 nm (31.9 Ω mm). However, as V_{GS} changed from −10.0 to 20.0 V, the output current for the triple-gate MOSFET without $L_{S/D\text{-}G}$ cannot be controlled well.
